# Supplementary material for: Digital health literacy is linked to attitudes regarding the ethical aspects of digital health among patients with dermatologic comorbidities
Source: PLoS One. 2025 Sep 5;20(9):e0330916. doi: 10.1371/journal.pone.0330916 (PMC12412967; doi:10.1371/journal.pone.0330916)
Supplement: S1 Appendix — Spanish and English versions. (PDF) [file pone.0330916.s001.pdf]

Bioethical attitudes regarding digital health questionnaire (BADH). Spanish version

Cuestionario de actitudes bioéticas en relación con la sanidad digital

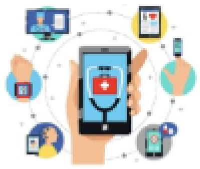

El presente cuestionario pretende conocer **su opinión** respecto al uso de las **tecnologías digitales o medios electrónicos** en algún aspecto de su **atención médica.**

Dentro de las **tecnologías digitales o (medios electrónicos)** se incluyen aplicaciones en computadoras o teléfonos celulares inteligentes, uso de internet, redes sociales, y expedientes médicos electrónicos.

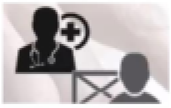

Por **cualquier aspecto de la atención médica** nos referimos a: agendar citas, consultar laboratorios, recibir recetas electrónicas, recibir consulta médica virtual o a distancia.

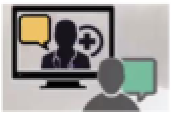

Las **consultas médicas virtuales (o a distancia** se pueden dar como una alternativa a las consultas presenciales y pueden requerir el compartir información personal o fotografías por vía electrónica ya sea mediante videoconferencias, correo electrónico o por teléfonos.

Seleccione con una cruz**, el espacio que corresponda con el grado en el que está en desacuerdo o de acuerdo con los siguientes enunciados. **Cero (0) En desacuerdo totalmente y Cuatro (4) De acuerdo totalmente.

	**En desacuerdo totalmente**	**Algo en desacuerdo**	**Indeciso**	**Algo de acuerdo**	**De acuerdo totalmente**
**1.** La información médica disponible en redes sociales es confiable.	0	1	2	3	4
**2.** Mi médico me atenderá mejor con la tecnología digital o medios electrónicos.	0	1	2	3	4
**3.** Durante mi atención médica, podré escoger usar o no tecnología digital.	0	1	2	3	4
**4.** Me preocupa que mi expediente electrónico se comparta sin mi autorización.	0	1	2	3	4
**En una consulta virtual:**<br>**5.** Podré opinar sobre mi tratamiento.	0	1	2	3	4
**En una consulta virtual:**<br>**6.** Las indicaciones médicas son confiables.	0	1	2	3	4
**En una consulta virtual:**<br>**7.** Me preocupa que personas diferentes a las que me atienden tengan acceso a mis datos personales.	0	1	2	3	4
**En una consulta virtual:**<br>**8.** Me preocupa que se compartan fotografías de mis lesiones sin mi autorización.	0	1	2	3	4

Bioethical attitudes regarding digital health questionnaire (BADH). English version

Questionnaire on bioethical attitudes in relation to digital health

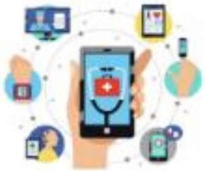

This questionnaire is intended to elicit **your opinion** regarding the use of digital technologies or **electronic media** in some aspect of your medical care.

Digital technologies or (electronic media) include applications on computers or smartphones, use of the internet, social networks, and electronic medical records.

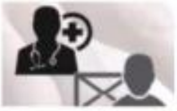

By **any aspect of medical care**, we mean: scheduling appointments, consulting labs, receiving electronic prescriptions, and receiving virtual or remote medical consultations.

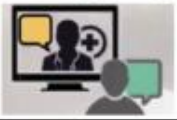

Virtual (or remote) medical consultations may be provided as an alternative to face-to-face consultations and may require the sharing of personal information or photographs electronically either by videoconferencing, email, or telephone.

***Select with a cross**, the space that corresponds to the degree to which you disagree or agree with the following statements. **Zero (0) Strongly Disagree and Four (4) Strongly Agree**.*

	Strongly Disagree	Some Disagree	Undecides	Some Agreement	Complete Agreement
**1.** The medical information available social media is reliable.	0	1	2	3	4
**2.** My doctor will treat me better with digital technology or electronic means.	0	1	2	3	4
**3.** During my medical care, I will have the choice to use or not use digital technology.	0	1	2	3	4
**4.** I am concerned that my electronic medical record may be shared without my consent.	0	1	2	3	4
**In a virtual consultation:**<br>**5.** I will be able to give my opinion about my treatment.	0	1	2	3	4
**In a virtual consultation:**<br>**6.** the medical indications are reliable.	0	1	2	3	4
**In a virtual consultation:**<br>**7.** I am worried that individuals other than those directly involved in my care may access my personal data.	0	1	2	3	4
**In a virtual consultation:**<br>**8.** I am concerned that photographs of my injuries may be shared without my consent.	0	1	2	3	4
