# Supplementary material for: Digital health literacy is linked to attitudes regarding the ethical aspects of digital health among patients with dermatologic comorbidities
Source: PLoS One. 2025 Sep 5;20(9):e0330916. doi: 10.1371/journal.pone.0330916 (PMC12412967; doi:10.1371/journal.pone.0330916)
Supplement: S1 Table — (PDF) [file pone.0330916.s002.pdf]

Supplementary Table 1. Comparison of socio-demographic characteristics between participants with high DHL levels and their counterparts.

	High-DHL patients n=31	Low-DHL patients n=89	p
Age, years	45 (32-58)	48 (33.5-62.5)	0.573
Females*	19 (61.3)	58 (65.2)	0.231
Years of education	17 (12-17)	12 (12-17)	**0.024**
Living with other people*	28 (90.3)	83 (93.3)	0.880
15-40 years old	17 (60.7)	52 (62.7)	1
41-60 years old	6 (21.4)	15 (18.1)	0.781
≥ 61 years old	22 (78.6)	56 (65.7)	0.342
Number of the people they live with[1]	3 (1-4)	2 (1-4)	0.841
Occupation*			
Formal and non-formal job	13 (41.9)	46 (51.7)	
Unemployed	8 (25)	9 (10.1)	
Housewife	5 (16.1)	22 (24.7)	0.151
Student	1 (3.2)	6 (6.7)	
Retired	4 (12.9)	6 (6.7)	
Patients benefiting from a regular monthly income*	17 (54.8)	60 (67.4)	0.260
Monthly income ≤ 500 USD[1]*	8 (47.1)	41 (68.3)	0.153

*Data presented as median (IQR) or otherwise indicated. *Number (%) of patients. [1]Among those with the characteristic.*
