# Supplementary material for: Digital health literacy is linked to attitudes regarding the ethical aspects of digital health among patients with dermatologic comorbidities
Source: PLoS One. 2025 Sep 5;20(9):e0330916. doi: 10.1371/journal.pone.0330916 (PMC12412967; doi:10.1371/journal.pone.0330916)
Supplement: S2 Table — (PDF) [file pone.0330916.s003.pdf]

Supplementary Table 2. Comparison of patients' digital access and usage characteristics between participants with high DHL levels and their counterparts.

	**High-DHL patients n=31**	**Low-DHL patients n=89**	**p**
Access to internet	29 (93.5)	77 (86.5)	0.516
Access to paid internet[1]	28 (96.6)	74 (96.1)	1
Full (every day) access to internet[1]	28 (96.6)	71 (92.2)	0.682
Good quality of internet connectivity	18 (62.1)	44 (57.1)	0.883
Availability of a mobile phone with internet access	30 (96.8)	88 (98.9)	0.452
Own mobile phone[1]	28 (93.3)	87 (98.9)	0.139
Availability of a computer	18 (58.1)	38 (42.7)	0.150
Own computer[1]	17 (94.4)	27 (71.1)	**0.079**
Availability of a tablet	6 (19.4)	5 (5.6)	**0.033**
Own tablet[1]	6 (100)	4 (80)	0.455
Availability of a smart watch	4 (12.9)	6 (6.7)	0.280
Use of email	29 (93.5)	69 (72.5)	**0.059**
Use of social media			
Whatsapp	29 (93.5)	83 (93.3)	1
Telegram	6 (19.4)	14 (15.7)	0.780
Facebook	22 (71)	65 (73)	0.819
X (formerly Twiter)	5 (16.1)	13 (14.6)	0.779
Youtube	22 (71)	61 (68.5)	1
Instagram	15 (48.4)	33 (37.1)	0.293
TikTok	10 (32.3)	32 (36)	0.828
LinkedIn	6 (16.1)	7 (7.9)	0.294
Other	0	0	0

Data presented as Number (%) of patients. [1]Among those with the characteristic.
