# Supplementary material for: Digital health literacy is linked to attitudes regarding the ethical aspects of digital health among patients with dermatologic comorbidities
Source: PLoS One. 2025 Sep 5;20(9):e0330916. doi: 10.1371/journal.pone.0330916 (PMC12412967; doi:10.1371/journal.pone.0330916)
Supplement: S3 Table — (PDF) [file pone.0330916.s004.pdf]

Supplementary Table 3. Comparison of factors related to the patients' dermatological diagnoses between participants with high DHL levels and their counterparts.

	High-DHL patients n=31	Low-DHL patients n=89	p
**Reason for dermatological consultation**			
Dry skin	11 (35.5)	29 (32.6)	0.826
Drug reaction	4 (12.9)	2 (2.2)	**0.038**
sunspots	6 (19.4)	18 (20.2)	1
Skin irritation/Allergies	10 (32.3)	18 (20.2)	0.218
Skin cancer	2 (6.5)	9 (10.1)	0.726
Nails changes	2 (6.5)	10 (11.2)	0.729
Urticaria (hives)	7 (22.6)	8 (9)	0.662
Acne	2 (6.5)	8 (9)	1
Mole revision	3 (9.7)	11 (12.4)	1
Other	12 (38.7)	32 (36)	1
**Lesions localization**			
Face	18 (58.1)	56 (62.9)	0.671
Genitals	4 (12.9)	13 (14.6)	1
Other areas of the body	19 (61.3)	61 (68.5)	0.510
**Comorbidities**			
Diabetes	6 (19.4)	16 (18)	1
Hypertension	7 (22.6)	22 (24.7)	1
Thyroid disorders	6 (19.4)	26 (29.2)	0.351
Cardiac disorders	5 (16.1)	2 (2.2)	**0.012**
Neurological conditions	4 (12.9)	8 (9)	0.505
Rheumatological conditions	6 (19.4)	22 (24.7)	0.628
Hematological conditions	2 (6.5)	5 (5.6)	1
Cancer	4 (12.9)	14 (15.7)	1
Transplant	3 (9.7)	14 (15.7)	0.553
HIV infection	3 (9.7)	9 (10.1)	1
Other	12 (38.7)	20 (22.5)	**0.099**

Data presented as Number (%) of patients. [1]Among those with the characteristic.
