# Supplementary material for: Digital health literacy is linked to attitudes regarding the ethical aspects of digital health among patients with dermatologic comorbidities
Source: PLoS One. 2025 Sep 5;20(9):e0330916. doi: 10.1371/journal.pone.0330916 (PMC12412967; doi:10.1371/journal.pone.0330916)
Supplement: Attachment — Submitted filename: RESPONSE TO REVIEWER.docx [file pone.0330916.s007.docx]

**RESPONSE TO REVIEWERS**

PONE-D-25-15276
Digital health literacy is linked to attitudes regarding the ethical aspects of digital health among patients with dermatologic comorbidities. PLOS ONE

**Comments from Editorial Office**

Thank you very much for submitting your manuscript to PLOS ONE, and for responding to our recent requests regarding your submission. After careful evaluation, we have decided that your submission does not meet our publication criteria and must be rejected.

Based on our evaluation of your manuscript and the information you provided, we do not feel that your submission meets our ethical requirements for human subjects research submissions. PLOS ONE requires that research meets all applicable standards for the ethics of experimentation and research integrity (<http://journals.plos.org/plosone/s/human-subjects-research>). We reserve the right to reject any submission that does not meet our internal ethical standards, which in some cases are more stringent than local ethical standards.

We recognize that you obtained ethical approval from your local ethics committee after you began your study: your ethics approval document is dated 28 February 2024, while you began collecting patient samples on 13 January 2024. You also state that informed consent was obtained from all participants, which indicates that the samples were prospectively collected for research purposes. For human subjects research, PLOS ONE requires that authors obtain ethics committee approval before beginning the study, and we do not accept retrospective ethical approval.

**Response**

Thank you for reviewing the article PONE-D-25-15276, titled “Digital Health Literacy is Linked to Attitudes Regarding the Ethical Aspects of Digital Health Among Patients with Dermatologic Comorbidities.”

We acknowledge our error and would like to clarify that it is common for our projects to involve patients as research partners during the development of the project idea before formal submission to the ethics committee. This initial engagement took place on January 13, 2024, when we met with several patients to explore the project concept. We believe that in this context, the role of the patient is that of a collaborator/research partner rather than a study participant. This approach has been suggested for implementation in the recent update of the Helsinki Declaration 2024. I would like to clarify that the involvement of patients at this stage does not necessarily fulfill the criteria for co-authorship. Once the project was thoroughly developed, it was submitted to the local IRB for evaluation and was approved on February 28, 2024. The first patient included in the study as a participant signed the informed consent on April 25, 2024.

As a research group, we are committed to adhering to good practices in human research and the highest ethical standards. We understand that the inclusion of patients must always occur after obtaining ethics committee approval, and we recognize that we failed to clarify the distinction between a patient functioning as a research partner (as occurred on January 13, 2024) and a patient functioning as a research participant (beginning April 25, 2024). We deeply regret this oversight but want to assure you that the study was conducted under stringent ethical standards, and recruitment began only after the approval date.

Finally, the Chairman of our Institutional Review Board has been informed about communications with the academic editor assigned by PLOS One.
