# Supplementary material for: Digital health literacy is linked to attitudes regarding the ethical aspects of digital health among patients with dermatologic comorbidities
Source: PLoS One. 2025 Sep 5;20(9):e0330916. doi: 10.1371/journal.pone.0330916 (PMC12412967; doi:10.1371/journal.pone.0330916)
Supplement: Attachment — Submitted filename: Comment from Reviewer for PONE-D-25-17804.docx [file pone.0330916.s008.docx]

**Comment from Reviewer**

Lovely article, short and sweet, all the important points are there.

Maybe add a bar chart or line graph to add some variation to the tables.

Well done to the authors.
