# Supplementary material for: Digital health literacy is linked to attitudes regarding the ethical aspects of digital health among patients with dermatologic comorbidities
Source: PLoS One. 2025 Sep 5;20(9):e0330916. doi: 10.1371/journal.pone.0330916 (PMC12412967; doi:10.1371/journal.pone.0330916)
Supplement: Attachment — Submitted filename: Bruce Review Comments for PLOS ONE Journal.docx [file pone.0330916.s009.docx]

**Bruce Review Comments for PLOS ONE Journal**

Abstract;Clarification and Precision

Line 22–23: Define "digital health literacy (DHL)" briefly. Readers unfamiliar with the term may benefit from a concise explanation.

Line 39: Instead of “p ≤ 0.0001”, revise to “p < 0.001” to align with APA and biomedical reporting conventions.

2. Introduction –Explicit Problem Statement and Context

Lines 105–106: The problem statement is implied but not clearly articulated. Add a focused paragraph stating the knowledge gap: e.g., “Despite growing attention to digital health tools, the ethical dimensions of patient engagement—particularly how DHL shapes moral attitudes remain understudied, especially in Latin American settings.”

Lines 108–109: Justify the focus on dermatologic patients more explicitly. Explain whether this population is particularly affected by digital health applications or ethical dilemmas in care.

3. Methodology – Sampling and Analysis Transparency

Lines 147–148: Clarify how the use of convenience sampling may limit generalizability. Acknowledge potential bias in participant selection.

Line 236: When discussing regression modeling, explain how multicollinearity was handled (e.g., via correlation matrix or VIF).

Lines 228–229: Justify the use of the 75th percentile as a cut-off for defining high DHL. Was this based on prior literature or purely exploratory?

4. Instrument Validation – Reliability Concerns

Line 292: The Cronbach’s alpha of 0.686 for the overall BADH is below the conventional threshold of 0.70. Acknowledge this as a limitation in tool reliability and consider recommending item revision or expansion in future studies.

Line 298: The privacy subscale shows moderate floor (13%) and ceiling (12%) effects. These should be addressed as potential threats to sensitivity and discussed in the limitations.

5. Results: Visual Clarity and Focus

Lines 311–317: The relationship between DHL and ethical attitudes is clearly reported but would benefit from a visual summary (e.g., scatterplot or regression line figure) to help readers interpret the statistical findings.

6. Discussion: Theoretical and Contextual Depth

Lines 342–344: The absence of association between DHL and privacy concerns deserves deeper theorization. Suggest discussing whether privacy concerns are more influenced by cultural distrust or systemic factors than by individual literacy levels.

Lines 464–467: While acknowledged, the sample size of 100 for factor analysis is relatively low. Consider citing literature supporting sufficiency or acknowledging this limitation more directly.

Line 472: The Latin American context is emphasized, but the policy and practical implications for digital health equity in the region are missing. Add a forward-looking statement linking findings to health policy or system-level change.

Conclusion: Strengthen Practical Implications

Line 480: Clarify that the BADH is “among the first validated instruments” for assessing ethical attitudes in digital health not necessarily the first ever.

Recommend adding actionable recommendations, such as incorporating DHL and ethical orientation training into patient education or clinician communication tools.
